# Fundamental limits in high-Q droplet microresonators

**DOI:** 10.1038/srep41997

**Published:** 2017-02-07

**Authors:** A. Giorgini, S. Avino, P. Malara, P. De Natale, G. Gagliardi

**Affiliations:** 1 Consiglio Nazionale delle Ricerche, Istituto Nazionale di Ottica (INO), via Campi Flegrei 34, Complesso “A. Olivetti”, 80078 Pozzuoli (Napoli), Italy; 2 Consiglio Nazionale delle Ricerche, Istituto Nazionale di Ottica (INO), Largo E. Fermi 6, 50125 Firenze, Italy

## Abstract

Liquid droplet whispering-gallery-mode microresonators open a new research frontier for sensing, optomechanics and photonic devices. At visible wavelengths, where most liquids are transparent, a major contribution to a droplet optical quality factor is expected theoretically from thermal surface distortions and capillary waves. Here, we investigate experimentally these predictions using transient cavity ring-down spectroscopy. With our scheme, the optical out-coupling and intrinsic loss are measured independently while any perturbation induced by thermal, acoustic and laser-frequency noise is avoided thanks to the ultra-short light-cavity interaction time. The measurements reveal a photon lifetime at least ten times longer than the thermal limit and indicate that capillary fluctuations activate surface scattering effects responsible for light coupling. This suggests that droplet microresonators are an ideal optical platform for ultra-sensitive spectroscopy of highly transparent liquid compounds in nano-liter volumes.

Dielectric micro-resonators obtained from solid glasses with different geometries have proven very promising devices for bio-chemical sensing^1^, optical-frequency comb generation^2^ and cavity opto-mechanics^3^. The sample delivery modalities and the weak interaction with the whispering-gallery-mode (WGM) evanescent tail severely limit their feasibility as sensors in liquid environments and integration in microfluidic systems. The possibility to form liquids into small, easy-to-handle droplet microresonators has triggered a strong scientific interest towards the creation of novel versatile photonic elements, i.e. a lab-in-a-droplet platform^4^,^5^,^6^,^7^. The long-standing issue of direct sensing in liquid media may be overcome in an elegant and effective way as a liquid resonator comprises the sensing unit and the sample under investigation at the same time. On the other hand, droplets are extremely appealing as opto-mechanical oscillators as they are among the simplest optofluidic devices and are made of materials 10^6^ times softer than glasses, which increase the amplitude of Brownian fluctuations and capillary waves^8^,^9^. Excitation of high-quality WGMs in droplets have been recently demonstrated and advanced techniques from the realm of standard cavities have been extended to liquids for lasing, spectroscopy, sensing and manipulation^10^,^11^,^12^,^13^,^14^,^15^,^16^. Analogously to solid microcavities, the quality factor is the primary figure of merit, which carries information on the photon lifetime within the system. The main limitations usually arise from material loss, scattering and coupling mechanism.

In this regard, besides absorption and scattering, also liquid surface tension is crucial to the optical features of droplets. Indeed, thermally-induced shape distortions restored by surface tension give rise to capillary waves on the surface that typically occur at frequencies in the 0.1–1 MHz range, depending on the size and material^17^,^18^. They manifest as a fast jitter of the optical resonances^18^,^19^ and thus inevitably affect the Q-factor when observed by a direct spectroscopic measurement. Surface light scattering is strongly influenced by capillary waves which can be used as a direct way to measure surface tension and viscosity of fluids^20^,^21^. In addition, similar effects may be caused by optical-to-mechanical energy transfer or by light impulses that induce acoustic waves via electrostriction^22^,^23^. It is still not obvious to what extent this kind of opto-mechanical feedback influences the Q-factor of microresonators and whether it is detrimental or not^18^. All these physical processes affecting the quality factor are not only crucial for characterization of droplet microresonators as liquid sensors but also for their use as miniaturized laboratories to study surface tension and rheological properties of materials via light scattering on the liquid-air interface^21^. In our work, we investigate experimentally the Q-factor of various oil-droplet microresonators with regard to the limits dictated by scattering, thermal noise and optical absorption loss, using a free-space excitation scheme to fully harness the benefits of a liquid cavity. We perform cavity ring-down measurements in its relaxation regime that provide information on the photon lifetime, and determine the effective Q-factor influenced by the coupling mechanism, i.e. the loaded Q-factor. We find that light scattering due to surface thermal capillary waves serves as an optical access to the whispering-gallery modes. We demonstrate that the Q-factor of highly-transparent, vertically-suspended oil spheres can be higher than 10^7^, in the visible region, revealing the intrinsic cavity photon lifetime that in most experiments is masked by thermal effects. Moreover, our results show that the ultimate Q-factor is not limited by thermal surface fluctuations as opposed to theoretical predictions.

## Results

### The optical system

**Figure 1:**
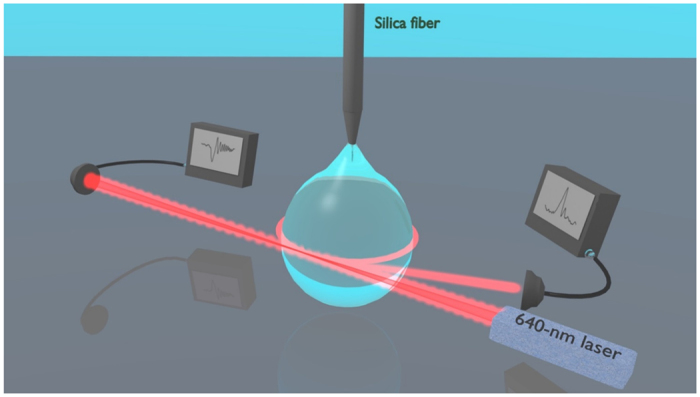
Experimental arrangement. A visible diode laser is focused close to the droplet rim to excite WGMs. Ringing signals are observed where directly-transmitted light and the scattered light are detected.

**Figure 2:**
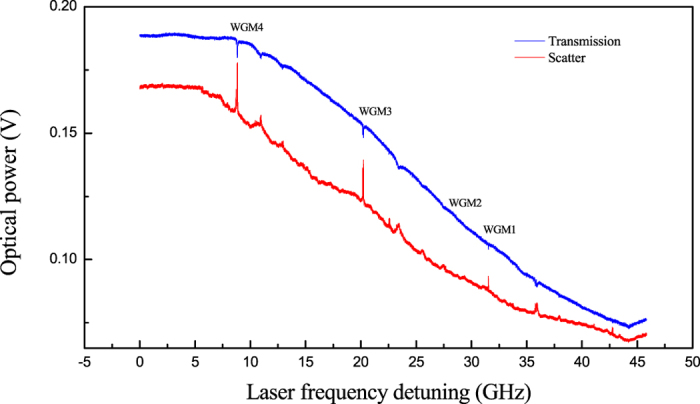
WGM excitation along a laser-frequency sweep (13-Hz frequency) for a paraffin droplet (300-μm radius). The three radial modes excited by scattering show different linewidths. Lower-Q modes are visible on the background.

Our experimental layout is shown in Fig. 1, where a liquid oil droplet, suspended by the tip of a silica fiber serves as the optical cavity that is interrogated by a visible diode laser. The pendant droplet whispering-gallery modes are directly excited by focusing the free-space laser beam tangential to the droplet surface, without using any coupling device (see Methods). In our configuration, the beam focus optimal position is just inside the surface edge and the cavity is undercoupled with 5–10% of the incident power effectively injected into low-order WGMs. Basically, the main optical coupling mechanisms are believed to be surface scattering^24^,^25^ and chaos-assisted tunneling^26^, whereas evanescent-wave coupling to WGM in free-space is not relevant due to the small droplet curvature^27^,^28^. Moreover, as remarked in ref. 27, van de Hulst’s localization principle fails in the case of a droplet with surface irregularities. Small irregularities caused by thermal capillary waves induce angular-momentum coupling of partially-scattered light waves to the resonator WGMs with optimal excitation efficiency for a light beam focused slightly inside the surface^29^,^30^, in agreement with experimental findings. Direct transmission and scattering WGMs are collected by additional lenses and focused on separate photodiodes. A current scan is sent to the laser to observe several WGM spectra. Figure 2 shows three of the narrowest resonances detected over a cavity free-spectral-range, where positive peaks correspond to the scattered light while negative dips appear in the transmitted light. The signal spectra are highly repeatable and the alignment of the droplet is not critical to the quality factor as opposed to tapered-fiber or prism coupling schemes^31^. Thanks to free-space beam illumination, a number of resonances are simultaneously excited in the liquid cavity, being likely associated to WGMs of low and high radial orders with narrower and wider lineshapes, respectively. In addition, the elliptical geometry of the suspended droplet potentially splits WGMs among different azimuthal orders^3^. A comprehensive theoretical description of the scattering efficiency and internal intensities for any dielectric sphere can be found in ref. 32, reproducing quite well the spectra that we experimentally observed. Droplet cavities made of other oils or water can be obtained with similar Q-factors but paraffin and silicone oil have been preferred for this study owing to their low evaporation rate and high transparency.

### Theoretical predictions

The Q-factor of the WGM of a droplet, with optical frequency ν, can be expressed in terms of the different loss channels that contribute to reduce the overall photon lifetime τ as^33^


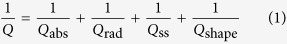


where *Q* = *2πντ* and *Q*_abs_, *Q*_rad_, *Q*_ss_, *Q*_shape_ are the contributions of material absorption, radiative loss, surface scattering loss and thermal shape distortions, respectively. A relative decay rate can be associated with each of the above terms. As remarked above, in microresonators with radii of the order of few hundreds μm, radiative loss is negligible compared to surface scattering loss, which in fact is the main in-out coupling mechanism. However, the factors related to absorption and thermal noise dominate the quality budget of our liquid microresonator. In particular, shape distortions due to thermally-induced capillary waves on the liquid surface have clearly been evidenced by current theoretical models as an inherent limitation to the observed Q-factor^18^. Indeed, they modulate the splitting of quasi-degenerate azimuthal modes due to a dynamical change in the droplet eccentricity thus leading to an effective WGM line broadening. The corresponding reduction of quality factor, considering the dependence of the azimuthal mode frequency on the droplet eccentricity and assuming a WGM with the lowest radial order and maximum angular momentum (*l* ≈ 2*πnα*/*λ*)^19^, is


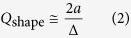


where ∆ is the root-mean-square amplitude of the thermally-induced droplet fluctuations. The latter is given by a recent theoretical study about surface displacements on a spherical liquid-air interface^34^


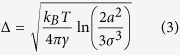


where *k*_*B*_, *γ*, and *T* are the Boltzmann constant, the liquid surface tension and the absolute temperature, respectively, while πσ^2^ is the effective area per molecule^34^. If we consider *a* = 450 μm as an average value within our size range, *T* = 296 *K, γ* = 0.026 N/m, and *σ* ≈ 6·10^−10^ m (calculated from the average molecular radius with a molecular weight ∼500 for oils), the limit set by thermal fluctuations in Eq. (2) would be 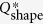 = 1.6∙10^6^ for paraffin and other mineral oils.

The material absorption contribution to the cavity Q-factor can instead be expressed as


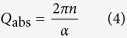


where *n* and *α* are the liquid refractive index and absorption coefficient at the laser wavelength *λ*. For paraffin and mineral oil (*n* = 1.47, *α* ≈ 0.7 m^−1^)^35^,^36^, *Q*_abs_ is expected to be ∼2·10^7^ while for silicone oil this is expected to be slightly higher owing to a very small optical absorption^10^. Heavy water (D_2_O) would exhibit even higher absorption-limited Q, having *α* ≈ 0.02 m^−1^ around 640 nm^37^.

### Lifetime measurements

Experimentally, the Q-factor of an optical cavity can be determined in different ways, the easiest of which relies on a spectroscopic measurement of the WGM optical frequency-to-linewidth ratio. However, this kind of measurement may be not fully accurate as it requires a non-linear fit of the WGM resonance profile that, particularly for Q-factors >10^6^, is seriously affected by laser-cavity frequency jitter and its conversion to amplitude noise, acoustic noise and photothermal broadening while it changes significantly with the observation time^27^,^38^,^39^. A time-domain measurement of photon lifetime provides instead a direct, reliable, very-low noise estimate of the overall cavity loss. This can be performed by cavity ring-down spectroscopy (CRDS), i.e. a measurement of the characteristic time of the exponential decay resulting from fast shutting a resonant laser beam^6^,^39^. However, a microresonator typically exhibits lifetimes in the order of 1–10 ns that makes ring-down measurements challenging even for high bandwidth modulation devices and detection electronics. Also, it only provides the loaded Q-factor value. An alternative method consists in the so-called rapidly-swept CRDS, where the laser is not constantly resonant whereas it is scanned through the resonance faster than the cavity build-up^39^,^40^. Because of the time-beating between photons entering the cavity and those previously stored into it, a chirped ringing response emerges on the transmitted beam that carries information on the scan rate, the intrinsic cavity lifetime and the coupling loss rate^41^. The analytical function of the microcavity transmission in a non-stationary condition is given by T 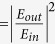 with *E*_*out*_ and *E*_*in*_ the output and input cavity fields, respectively, which are related each other by^42^


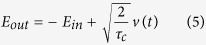


and


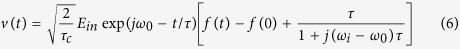



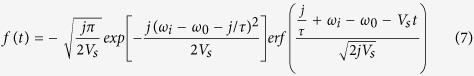


where erf (z) with z ∈ C denotes the complex error function. Here 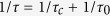 is the loaded photon lifetime, where *τ*_*c*_ is the loss rate associated with the out-coupling and *τ*_*c*_ is the intrinsic photon lifetime.

**Figure 3:**
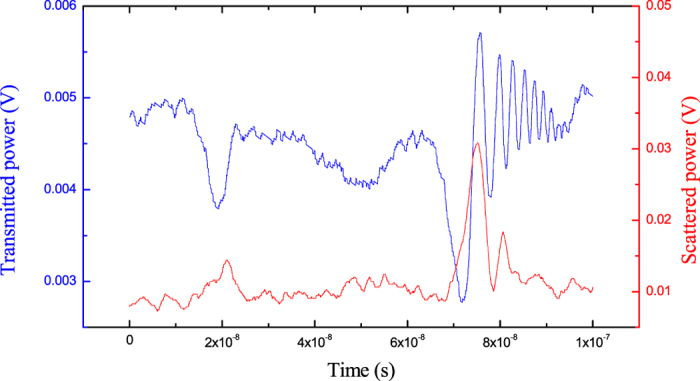
Transient ringing waveforms recorded during a single, very-fast linear laser sweep (∼10^17^ Hz/s) across the resonances corresponding to WGM1 and the weak mode WGM2.

**Figure 4:**
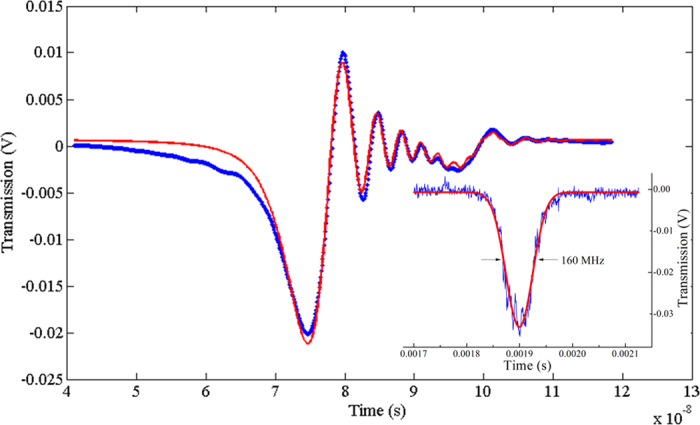
Fitting of the WGM3 resonance with Eq. 5 (red line) after subtraction of the linear power background and 4000-waveform averaging. The overall lifetime is τ_3_ = 4.8 ± 0.1 ns (errors are 1σ standard deviation; time resolution is 5 ps). The inset shows a recording in the spectral domain performed by a slow laser-frequency scan (transit time through resonance ~0.4 ms) along with a Gaussian fit (red line): the measured FWHM is 160 ± 1 MHz (Q = 2.93 ± 0.02·10^6^).

In Fig. 3, we show the ringing profiles corresponding to the sharpest resonance on the blue side of Fig. 2 (WGM1) and a neighboring peak (WGM2), as recorded from the droplet transmitted and scattered power (see Methods). As expected, distinct oscillations appear only on the narrowest peak (WGM1). It is worth noting that the scattered power and the transmitted power exhibit a different symmetry in their ringing profiles^40^. Also, the ringing signal of WGM3 after multiple-waveform averaging is shown in Fig. 4 (see Methods). The averaged time-domain spectra of all WGMs are fitted with the expressions of Eqs (5)–(7), 12, 9 retrieving lifetimes *τ*_1_ = 6.35 ± 0.05 ns (Q_1_ = (1.87 ± 0.01) 10^7^) for the narrowest mode and *τ*_3_ = 4.8 ± 0.1 ns (Q_3_ = (1.41 ± 0.03) 10^7^) for the central mode. As an example, in Fig. 4, we show the agreement between the experimental data and a multiple-resonance fit curve that accounts also for the presence of a weak mode nearby. The resulting values are about one order of magnitude larger than the calculated thermal-noise bound 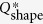. We have repeated the same measurements varying the beam-droplet distance within a reasonable resonance-visibility range (±2.5 μm) without finding any significant change in the lifetimes.

**Figure 5:**
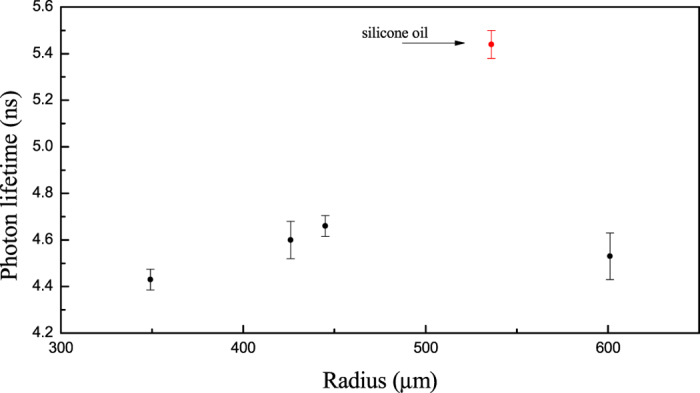
Lifetime values extracted from the fit of ringing waveforms of paraffin droplets with different radii (the red point corresponds to a silicone oil droplet).

The large measured Q-factors are far beyond the expectations based on liquid paraffin absorption only. To further verify this hypothesis, we have suspended four paraffin-oil droplets with different radii and performed subsequent measurements with the time-ringing technique on the central mode, chosen as the best compromise in terms of signal-to-noise ratio and linewidth. The measured decay times fall between 4.43 ± 0.04 ns and 4.66 ± 0.05 ns, as shown in Fig. 5, leading to Q-factors between (1.30 ± 0.01) 10^7^ and (1.37 ± 0.01) 10^7^. From this measurement set, we note that the lifetime remains constant with the drop radius within 2σ, even when it is doubled. The τ value changes instead if a different liquid is used, with 5.44 ± 0.05 ns (Q_s_ = (1.60 ± 0.01) 10^7^) for silicone oil. This argument leads us to conclude that the quality factor is mainly affected by intra-cavity absorption loss. Also it confirms that thermal noise is not the actual limit since the dependence on the droplet radius expressed in Eq. (2) is not found in the experiment.

On the contrary, the quality factor *Q* = *ν*/*Δν* seen from the spectral width *Δν* of WGM resonances in a stationary condition with a much lower scan rate (0.5-ms transit through resonance) is found to be ∼3·10^6^, as shown by the Gaussian fit of WGM3 in the inset of Fig. 4. This value is close to the limit predicted by Eq. (2) and Eq. (3). The discrepancy with the time-domain measurements can be understood considering that thermally-induced shape distortions do not act on the ns-scale ringing signals which thus exhibit a longer photon lifetime, as results from fitting with Eqs (5)–(7), 12, 9. On intermediate timescales (50–0.5 μs), slightly larger Q-factors are observed (between 4·10^6^ and 5·10^6^) but the resonant lineshapes start showing hybrid features between the ringing and the stationary profile that are difficult to analyze and fit. If on one hand this behaviour prevents a reliable linewidth assessment, on the other it strongly indicates that thermally-induced fluctuations act on a timescale in the order of 10^−6^–10^−5^ s^16^,^17^.

It is also worth noting that all the fits consistently yield a value of *τ*_*c*_ ≈ 7 μs, i.e. >1500 times the intrinsic lifetime *τ*_*0*_, that points out the droplet cavity does not suffer from any loading effect in the case of free-space excitation. We remark that the decay rate 1/*τ*_*c*_ provides a direct estimate of the out-coupling loss, i.e. the scattering loss. This contribution is likely due to the average amplitude of thermally-induced broad-spectrum capillary waves on the droplet surface. The surface scattering factor can be calculated with the formula^43^


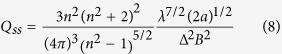


proposed and experimentally tested in [43] for solid microresonators in the 600-nm wavelength range, where *n* is the liquid refractive index and *B* is the surface-irregularity correlation length. Taking *B* ∼ 6 nm^44^ and the above found value of Δ, we obtain *Q*_*SS*_ ∼ 2·10^10^ corresponding to a lifetime *Q*_*SS*_/2π ∼ 6.9 μs, in satisfactory agreement with the value of *τ*_*c*_ retrieved from our ringing measurements.

**Figure 6:**
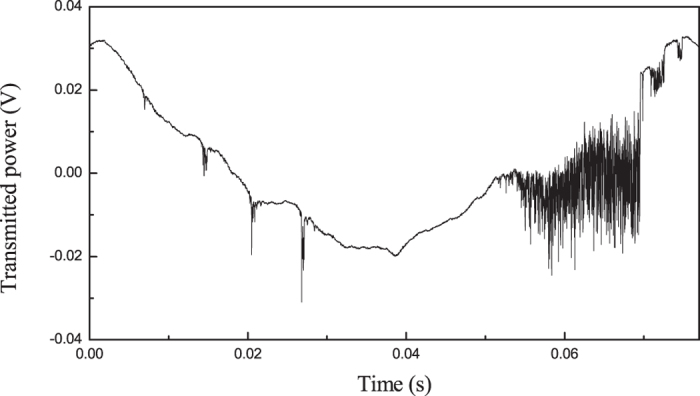
Thermal broadening/narrowing effect and hysteretic response of a WGM resonance as seen on both sides of a slow linear wavelength scan (scan range ∼1 GHz).

In principle, other physical mechanisms might be playing a role in the microresonator loss budget. According to the general theory^18^, the resonance linewidth may deviate from the thermal-noise limit of Eq. (3) due to imperfect excitation of the spectral multiplet within the WGM or in the presence of non-linear scattering phenomena that may even cause line-narrowing. While we doubt that only a partial overlap of the laser with the WGM occurs in our case, the second scenario is possible in principle. In Fig. 6, we show the very strong thermally-induced nonlinearity observed when scanning the laser across a WGM, which points out a hysteretic behaviour on the ascending/descending sides of the scan^27^. Strong frequency fluctuations are visible on the resonance lineshape. These effects are more and more relevant when going to slower scan speeds and eventually prevents us to take WGM spectra for linewidth measurements longer than few ms at full laser power. In order to find out whether such strong energy transfer to the cavity is accompanied by non-linear variations of the Q-factor, we have performed a new set of ringing cavity ring-down measurements by gradually decreasing the incident power down to one order of magnitude. However, no correlated change was observed in the photon lifetime. Next, we have frequency-locked the laser on one of the narrowest resonances using an all-optical feedback loop^13^ and we have calculated the fast-Fourier transform (FFT) of the transmission and scattering signals. From the FFT noise spectra only an overall increase in the amplitude noise is found on resonance without any noticeable spectral feature associated with high-frequency vibrations or surface waves. Therefore, no conclusive evidence of non-linear optical effects in liquid droplets has been found.

## Discussion

Our experimental results provide evidence that, on a time-scale shorter than thermal fluctuations, the Q-factor of free-space coupled liquid microresonators can be as high as their solid counterpart. Despite theoretical models, we experimentally demonstrate that the actual limitation to the photon lifetime is material absorption, which points to a quality factor >10^8^ for highly transparent liquids. Indeed, when observing the laser transit through resonance along a timescale shorter than that of typical cavity distortions, a measurement of the ringing decay discloses the intrinsic cavity lifetime *unaffected* by thermal and mechanical noise. In this way, the measured Q-factor corresponds to the true lifetime instead of an effective one. This favorable condition can be readily achieved for droplets with diameters up to 1 mm, which typically show photon lifetimes much shorter than 10^–6^ s even for very high quality factors. We find in turn that surface scattering due to thermally-induced fluctuations plays a crucial role for light coupling and we confirm that large beam misalignments along with power or size changes do not affect coupling efficiency. This suggests that droplet microresonators can be a convenient and robust optical platform for spectroscopy and bio-sensing in the liquid phase. In particular, this approach is of great potential for the measurement of ultra-low absorbing liquid compounds in sub-μl size samples in a given wavelength range, e.g. water in the visible^37^, where conventional methods are not easily applicable. As a future prospect, investigations on Brownian motion, viscoelasticity, droplet coalescence and surface capillarity phenomena in a controlled environment will be possible^21^,^45^,^46^.

## Methods

As shown in Fig. 1, a distributed-feedback (DFB) diode laser, emitting at a wavelength of 640 nm, is used to interrogate the liquid cavities. The laser is driven by a precision temperature controller and a low-noise current generator. A paraffin or silicone oil droplet, suspended by the tip of an acrylate-coated silica fiber (250-μm outer diameter) owing to surface tension^6^, serves as the optical cavity. Its radius *a* ranges approximately from 200 μm to 600 μm depending on the minimum volume required for a stable, symmetrical droplet and the maximum weight allowed by the equilibrium between surface tension and gravity force. A microscope objective tightly focuses the visible laser beam tangential to the droplet rim, in an equatorial plane, in order to excite WGMs. The resonance spectra are calibrated using a high-finesse Fabry-Perot interferometer with a 1-GHz free-spectral-range. A fine alignment of the droplet into the light beam is done by a piezo-actuated xyz translation stage with 20-nm resolution. The laser source is wavelength tuned via the current generator at frequencies up to 1 MHz and can be precisely locked to a single WGM resonance. For this purpose, a servo amplifier is used as a feedback element to control the laser wavelength. A suitable frequency discriminator to drive the servo input can easily be obtained from the far-field spatial mode interference between scattered WGMs without resorting to conventional radio-frequency modulation schemes^13^. To observe the cavity ringing signals (Fig. 3), the optical power directly-transmitted and scattered by the droplet are detected by a 12-GHz photodiode and acquired by a fast oscilloscope (4-GHz bandwidth) while linear wavelength sweeps with 1-MHz frequency are applied to the laser injection current. The experimental spectra are first normalized by the laser-scan intensity slope and then averaged over 4000 fast-repeated acquisitions (with 300-ns time gap). The resulting curves, as shown in Fig. 4, are fitted with the expressions of Eqs (5)–(7), 12, 9 to extract the photon lifetimes.

## Additional Information

**How to cite this article:** Giorgini, A. *et al*. Fundamental limits in high-Q droplet microresonators. *Sci. Rep.* **7**, 41997; doi: 10.1038/srep41997 (2017).

**Publisher's note:** Springer Nature remains neutral with regard to jurisdictional claims in published maps and institutional affiliations.
